# The impact of aging and oxidative stress in metabolic and nervous system disorders: programmed cell death and molecular signal transduction crosstalk

**DOI:** 10.3389/fimmu.2023.1273570

**Published:** 2023-11-08

**Authors:** Kenneth Maiese

**Affiliations:** Innovation and Commercialization, National Institutes of Health, Bethesda, MD, United States

**Keywords:** AMPK, APOE-ε4, autophagy, diabetes mellitus, ferroptosis, pyroptosis, SIRT1, WISP1

## Abstract

Life expectancy is increasing throughout the world and coincides with a rise in non-communicable diseases (NCDs), especially for metabolic disease that includes diabetes mellitus (DM) and neurodegenerative disorders. The debilitating effects of metabolic disorders influence the entire body and significantly affect the nervous system impacting greater than one billion people with disability in the peripheral nervous system as well as with cognitive loss, now the seventh leading cause of death worldwide. Metabolic disorders, such as DM, and neurologic disease remain a significant challenge for the treatment and care of individuals since present therapies may limit symptoms but do not halt overall disease progression. These clinical challenges to address the interplay between metabolic and neurodegenerative disorders warrant innovative strategies that can focus upon the underlying mechanisms of aging-related disorders, oxidative stress, cell senescence, and cell death. Programmed cell death pathways that involve autophagy, apoptosis, ferroptosis, and pyroptosis can play a critical role in metabolic and neurodegenerative disorders and oversee processes that include insulin resistance, β-cell function, mitochondrial integrity, reactive oxygen species release, and inflammatory cell activation. The silent mating type information regulation 2 homolog 1 *(Saccharomyces cerevisiae*) (SIRT1), AMP activated protein kinase (AMPK), and Wnt1 inducible signaling pathway protein 1 (WISP1) are novel targets that can oversee programmed cell death pathways tied to β-nicotinamide adenine dinucleotide (NAD^+^), nicotinamide, apolipoprotein E (APOE), severe acute respiratory syndrome (SARS-CoV-2) exposure with coronavirus disease 2019 (COVID-19), and trophic factors, such as erythropoietin (EPO). The pathways of programmed cell death, SIRT1, AMPK, and WISP1 offer exciting prospects for maintaining metabolic homeostasis and nervous system function that can be compromised during aging-related disorders and lead to cognitive impairment, but these pathways have dual roles in determining the ultimate fate of cells and organ systems that warrant thoughtful insight into complex autofeedback mechanisms.

## 1 Introduction

Disorders such as diabetes mellitus (DM) and cellular metabolic disease are increasing in prevalence throughout the world. Over a thirty-five year course from the year 1980, the number of individuals with DM increased from one hundred eight million to over four hundred twenty-two million individuals (1, 2). By the year 2045, seven hundred million individuals may have DM (3, 4). From the years 2013 to 2016, the prevalence of DM has risen from over nine percent (5). DM is a chronic disorder that affects all organs of the body leading to cardiac disease, retinal disease, hepatic injury, cerebral ischemia, limb amputation, and renal failure (5–18). Almost one half billion individuals have DM and at least half of the four million deaths that occur per year with DM impact individuals less than seventy years of age (1, 7, 19–22). Ten percent of the population in the United States (US) are currently reported to suffer from DM (23, 24). However, it is believed that many additional individuals have disorders of metabolism or have elevated risk to develop DM, but remain undiagnosed at present (3, 16, 21, 25–37). It is estimated that in individuals greater than eighteen years old, seven million may not be correctly diagnosed as having DM and more than thirty-five percent of US adults may have prediabetes due to elevations in their fasting glucose and hemoglobin A1c (HbA_1c_) parameters (7, 38). Globally, four hundred million individuals are estimated to have metabolic disease or be at risk for developing DM (3, 39–41).

Metabolic disease affects low and middle income countries more than high income developed countries with approximately eighty percent of people residing in low-income nations (3, 42). This may be a result of the prevalence of DM being affected by a number of parameters that include socioeconomic status, comorbidities such as infection with the severe acute respiratory syndrome coronavirus (SARS-CoV-2), and level of education (43–54). In regard to education level, those individuals with less than a high school education represent thirteen percent of DM patients, individuals with a high school education equal ten percent of DM patients, and those individuals with more than a high school education represent approximately seven percent of DM patients (2). Other factors that can contribute to the development and progression of DM include limited exercise, tobacco consumption, high serum cholesterol, hypertension, and obesity (8, 13, 48, 55–57). Obesity alters a number of pathways in the body and can impact oxidative stress cell injury, stem cell survival, inflammation, aging processes, and the maintenance of mitochondrial function (21, 33, 40, 51, 56, 58–73). As a result, the additional body weight fosters insulin insensitivity and glucose intolerance that progresses to DM (19, 24, 28, 36, 74–80) (**Table 1**).

**Table 1:** Highlights The Impact of Aging and Oxidative Stress in Metabolic and Nervous System Disorders: Programmed Cell Death and Molecular Signal Transduction Crosstalk.

•With the increase in global lifespan and non-communicable diseases, metabolic disease affects low and middle income countries more than high income developed countries and by the year 2045, it is estimated that over seven hundred million individuals will have diabetes mellitus (DM).
•Metabolic disorders are intimately tied to the development and progression of neurodegenerative disorders that comprise over six hundred disease entities, impact greater than one billion people, and can lead to dementia as the 7^th^ leading cause of death.
•Aging processes, oxidative stress, dysfunction in telomere processing, and cell senescence are underlying mechanisms for the progression of metabolic disorders and neurodegenerative disease.
•Programmed cell death pathways of autophagy, apoptosis, ferroptosis, and pyroptosis can oversee a number of critical cellular functions that include reactive oxygen species (ROS) generation, the proliferation and size of pancreatic β-cells, insulin resistance, mitochondrial integrity, β-amyloid (Aβ) and tau brain deposition, and inflammatory cell activation.
•The silent mating type information regulation 2 homolog 1 *(Saccharomyces cerevisiae*) (SIRT1), AMP activated protein kinase (AMPK), and Wnt1 inducible signaling pathway protein 1 (WISP1) are novel targets that can oversee programmed cell death pathways tied to β-nicotinamide adenine dinucleotide (NAD^+^), nicotinamide, apolipoprotein E (APOE), severe acute respiratory syndrome (SARS-CoV-2) exposure with coronavirus disease 2019 (COVID-19), and trophic factors, such as erythropoietin (EPO) that can oversee these pathways such as with SIRT1 and AMPK.
•The pathways of programmed cell death, SIRT1, AMPK, and WISP1 offer exciting insights for maintaining metabolic homeostasis and neurovascular cell integrity that can be compromised during aging-related processes that can lead to cognitive loss, but these pathways have dual roles in determining the ultimate fate of cells and organ function that can have complex autofeedback mechanisms.

Additional challenges for the care of individuals with DM involve financial expenditures. At least twenty thousand United States Dollars (USD) on an annual basis is necessary for the basic care of people with DM that can involve the maintenance of glucose homeostasis, wound care, and nutritional education (6, 9, 19, 20, 28, 41, 74, 81–87). Yet, the required resources for DM care is growing and is greater than seven hundred sixty billion USD with an additional seventy billion USD necessary for those with severe disability and loss of function (3). Greater than seventeen percent of the Gross Domestic Product in the US is consumed for the care of people with DM (88).

**Figure 1:**
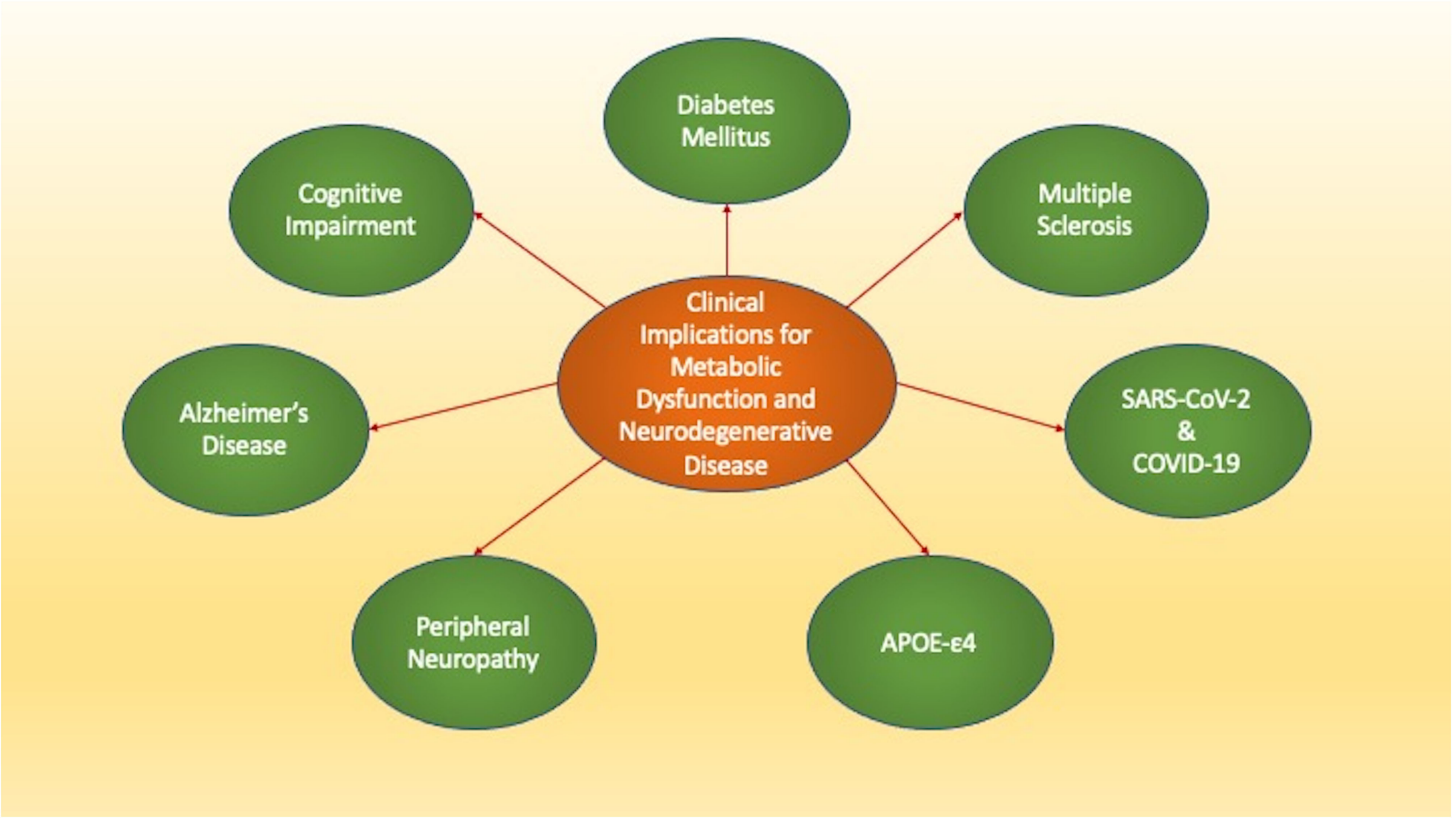
The Clinical Implications of Metabolic Dysfunction and Neurodegenerative Disease. Loss of metabolic homeostasis can lead to multiple disorders. Metabolic disorders affect both the peripheral and central nervous systems and can be affected by several risk factors. These disorders include diabetes mellitus that affects all systems of the body, cognitive impairment, Alzheimer’s disease, Multiple Sclerosis, and peripheral neuropathies. Additional entities such as the apolipoprotein E (APOE-ε4) gene, severe acute respiratory syndrome coronavirus (SARS-CoV-2), and coronavirus disease 2019 (COVID-19) can lead to memory loss, cognitive failure, and cortical vascular disease.

The debilitating nature of DM affects the entire body and leads to the degeneration of all organ systems (6, 7, 12, 16, 19, 21, 24, 27, 31, 34, 36, 46, 60, 85, 89–94). In particular, the metabolic disorders affect the nervous system and can lead to cognitive impairment, peripheral neuropathies, demyelinating disorders, and risk for developing infection as well as memory loss (**Figure 1**). An additional risk that includes metabolic disease for the development of neurodegenerative disorders is the observed rise in lifespan (95–100). Life expectancy is increasing especially in developed nations (101) and over the past fifty years the number of people greater than the age of sixty-five has increased greater than one hundred percent (4, 68, 96, 97, 102–113). Neurodegenerative disorders comprise a portion of non-communicable diseases (NCDs) and over seventy to seventy-five percent of the deaths that occur each year are due to NCDs (8, 22, 56, 60, 114–116). The increase in lifespan coincides with the rise of NCDs (91, 111, 117–129). As a result, the increase in lifespan for the world’s population has resulted in an increased prevalence for diseases of the nervous system (117, 130–133). Nervous system disorders comprise greater than six hundred disease entities, lead to the death of over seven million people annually, and can impact greater than one billion people (111, 117, 134–146). In relation to financial exposure, more than eight hundred billion USD in the US is required annually to care for multiple neurological disorders that include stroke, trauma, epilepsy, back pain, Parkinson’s disease (PD), Huntington’s disease (HD), amyotrophic lateral sclerosis (ALS), and dementia (116). Cognitive loss can be the most significant burden to the financial system and these cost considerations do not include the expenses required for companion care, social health programs, and senior daily care with the additional greater than seventy million clinicians and social workers needed to fill these unmet needs (22, 116, 147). These additional services will reach 2 trillion USD annually in the US and more than four million people will need over four billion USD for treatment each year. The market for dementia could exceed eleven billion USD (34, 141, 148). Furthermore, additional significant costs involve other neurological disorders, such as PD with greater than fifty-five billion USD necessary for care in the US annually. In the year 2030, the number of those affected with PD is predicted to double. Present expenses are currently at a large annual cost per individual of approximately twenty-five thousand USD per year (5, 97, 137, 144, 146, 149–165).

## 2 The intimate relationship among metabolic disease, oxidative stress, aging, and neurodegenerative disorders

Metabolic disorders, such as DM, increase the risk for the onset and progression of neurodegenerative disorders through multiple pathways. DM is a primary mechanism for the onset of cardiovascular disease that can ultimately lead to disorders of the nervous system (8, 53, 166–170). When compared to people that do not have DM, individuals with DM can have two times the risk of developing cardiac disability or cerebral ischemia (40, 43, 91, 108, 111). DM results in insulin resistance (9, 19, 24, 60, 80, 90, 171–174), vascular injury (16, 19, 26, 27, 29, 30, 32, 33, 61, 74, 86, 87, 170, 175–182), alterations in cerebral blood flow (2, 7, 9, 53, 91, 176, 183), endothelial dysfunction (19, 27, 40, 83, 94, 184), mitochondrial injury (13, 28, 53, 60, 115, 172, 178, 185, 186), retinal disease (30, 66, 94, 187–189), stem cell loss (35, 38, 54, 66, 72, 77, 84, 111, 190), susceptibility to infections (34, 46, 48–51, 54, 92, 191, 192), and immune system dysfunction (30, 54, 64, 68, 84, 173, 178, 193–199).

DM and cellular metabolism also play a significant role in the processes of oxidative stress and aging (7, 16, 47, 64, 75, 89–91, 108, 178, 196, 200). DM can lead to changes in transcriptional networks, loss of mitochondrial homeostasis, inflammation, production of reactive oxygen species (ROS), and cell senescence (7, 16, 20, 21, 30, 32, 66, 78, 79, 90, 91, 108, 174, 178, 193, 194, 201–203). ROS that are generated during oxidative stress include hydrogen peroxide, superoxide free radicals, nitric oxide, singlet oxygen, and peroxynitrite (36, 37, 65, 90, 96, 113, 120, 137, 154, 180, 186, 204–218). During conditions that oversee the detrimental effects of ROS, antioxidant systems are in play that involve glutathione peroxidase, catalase, superoxide dismutase, and the nutrient vitamins B, K, E, D, and C (20, 34, 42, 47, 53, 100, 132, 186, 202, 217, 219–225). If these systems are overwhelmed or unable to limit excessive ROS production, mitochondrial injury, loss of DNA integrity, and shortened lifespan can occur (8, 34, 69, 78, 96, 104, 120, 122, 162, 207, 209, 218, 226–229). Oxidative stress can result in vascular endothelial cell injury (9, 40, 83, 230–234), neuronal cell compromise (24, 66, 122, 123, 152, 156, 162, 233, 235–245), alterations in neurotransmitters (69, 246, 247), myelin degradation (79, 224, 248–252), cell senescence (8, 33, 55, 112, 253–255), loss of stem cell proliferation (34, 66, 168, 228, 241, 251, 256–259), and cognitive impairment (7, 10, 120, 121, 200, 223, 243, 245, 260–266).

In regard to aging and cellular metabolism, the shortening of telomeres (TLs), complexes of deoxyribonucleic acid (DNA), can lead to the increased risk for the development of DM (8, 267) and greater cell senescence with the loss metabolic homeostasis (16, 33, 178, 190, 268). Changes in TL length can promote aging processes, cellular senescence, and neurodegeneration as well (70, 112, 113, 118, 154, 255, 269–275). TLs are positioned on chromosome ends and oversee replication of cells, preservation of the genomic DNA, and cell survival (8, 16, 274, 276–279). More than two thousand repetitions of double-stranded non-coding DNA with the ‘TTAGGG” sequence that is finalized with guanine rich single-stranded DNA compose TLs (8, 70, 139, 265, 279). Complexes of proteins that include CTC1-STN1-TEN1 (CST), shelterin, and telosome are part of the TL family (7, 139, 252). To oversee the division of cells, these proteins control function and stability of TLs that can lose twenty-five to over two hundred base pairs during the process of dividing cells. Telomerase protein can prevent the loss of base pairs in TLs by providing tandem repeat ribonucleic acid (RNA) templates (274, 276, 280). However, cell senescence ultimately ensues when TLs become very short with less than five hundred base pairs and telomerase function is impaired (33, 95, 102, 118, 134, 154, 221, 253, 255, 269, 272, 275, 281). At this point, tissues and organs cannot undergo repair, the immune system is less viable, and age-related disorders can progress (7, 73, 139, 164, 282–286). These events with the shortening of TLs and cellular senescence also promote oxidative stress and the release of ROS that impairs cell organelles, such as mitochondria and cellular energy homeostasis (7, 32, 104, 121, 122, 196, 282, 287, 288).

## 3 The clinical onset of metabolic mediated neurodegenerative disease

Given the ability of DM to lead to aging processes tied to oxidative stress, neuronal and vascular injury, mitochondrial dysfunction, stem cell loss, and immune system disorders, it becomes evident that loss of metabolic homeostasis with DM can result in multiple neurodegenerative disorders. Metabolic disorders can affect both the peripheral and central nervous systems (**Figure 1**). In the peripheral nervous system in the presence of DM, autonomic dysfunction (289–291) and neuropathies can be common and affect more than seventy-five percent of individuals (5, 38, 78, 79, 83, 290, 292).

In the central nervous system, cognitive loss with DM is a significant co-morbidity. DM results in memory impairment (7, 10, 55, 115, 200, 263, 293–296) and can lead to the onset and progression of Alzheimer’s disease (AD) (2, 6, 28, 44, 89, 153, 201, 266, 297–302) (**Figure 1**). Dementia is present in all nations throughout the world and is now considered to be the seventh primary reason for death (109, 116, 131, 141, 144, 161, 164, 223, 252, 266, 303–308). At least five percent of the world’s population has dementia and by the year 2050 it is believed that over one hundred fifty-five million people will have cognitive loss (2, 73, 97, 115, 126, 205, 226, 275, 309, 310). Of those individuals with dementia, approximately sixty percent of people have the sporadic form of AD and greater than ten percent are over the age of sixty-five (5, 110, 139, 148, 277, 311–313). Diagnosis for dementia can fall significantly behind the onset of the disorder and may not be recognized until twelve to twenty-four months after the initial clinical presentation (38, 252, 314). Currently, more than six million people in the US have the sporadic form of AD (5, 309, 315–318), but this is expected to increase to thirty million people during the next two decades (6, 7, 110, 205, 226, 245, 282, 319, 320). In contrast, familial AD (FAD) is present in about two hundred families in the world (6, 73, 110, 121, 134, 282, 311). FAD represents an autosomal dominant version of amyloid precursor protein (APP) gene that is mutated, occurs in variable single-gene mutations on chromosomes 1, 14, and 21, and is usually clinical present prior to fifty-five years of age (115, 321, 322).

Risk factors also exist for dementia that can have a metabolic basis as well. In experimental models, insulin signaling can be associated with AD pathology (71). Late-onset AD can result in the presence of the ϵ4 allele of the apolipoprotein E (APOE-ε4) gene (5, 7, 148, 161, 252, 323–325) (**Figure 1**). The risk for developing AD is more than twenty times greater in those individuals with two APOE-ϵ4 alleles. APOE is produced in liver cells and is vital for metabolic cellular function to oversee the homeostasis of lipids through the transport of triglycerides, phospholipids, and cholesterol (7, 127, 161, 325–328). In the brain, astrocytes produce APOE to modulate the transfer of cholesterol to neurons through APOE receptors (7, 127, 252, 326, 328, 329). Interestingly, β-amyloid (Aβ) can be removed and destroyed by APOE through apoptosis and the exposure of phosphatidylserine (PS) membranes that are a part of the apoptotic cell death process (330, 331). Other forms of APOE that do not involve APOE-ε4 may inhibit Aβ aggregation during PS membrane exposure (332). However, Aβ aggregation is not believed to be blocked by APOE-ϵ4 which can therefore allow amyloid deposition to proceed and potentially foster the development of AD (44, 161, 323, 332–334). APOE-ϵ4 also may assist with the infection of viral antigens and lead to cerebral microhemorrhages during severe acute respiratory syndrome (SARS-CoV-2) exposure with coronavirus disease 2019 (COVID-19) (326) (**Figure 1**). SARS-CoV-2, a β-coronavirus family virion, has resulted in a global pandemic (34, 46, 49, 50, 335, 336) and can attach to nasal epithelial cells (337) and neurovascular cells in the brain (54). These processes subsequently result in hyperactivation of the immune system (335, 336, 338–340). Following SARS-CoV-2 infection, memory loss and cognitive failure can develop and lead to long-COVID, also termed long-haul COVID, chronic COVID-19, or post-acute COVID (34, 49, 50, 325, 336, 341–345). The combination of APOE-ϵ4 and SARS-CoV-2 infection can lead to cognitive loss and cortical vascular disease (57, 97, 181, 226, 326, 346–350).

Multiple sclerosis (MS), an additional significant neurodegenerative disorder, also may develop as a result of metabolic disease, pathways of APOE-ϵ4, and Aβ deposition (125, 252, 350–359) (**Figure 1**). MS impacts large portions of the global population, affects greater than two and one-half million people, and is a primary disease of myelin and myelin producing cells that is immune system mediated (159, 351, 358, 360–364). MS appears to lead to disease in more women than men (357) and can markedly impair cognitive function (252, 365, 366). The memory loss can be progressive in nature and affect both women and men (367). At least sixty-five percent of patients with MS have difficulty with memory recall and executive ability (252). Cognitive loss in MS patients may be tied to metabolic pathways and APOE-ϵ4 since individuals with MS can demonstrate lower cognitive function and delayed responses to stimuli (368). APOE serum levels are elevated in individuals with demyelinating optic neuritis and the genotype of *APOE* ϵ3/ϵ3 may lead to the male onset of optic neuritis (327). In the setting of APOE risk factors and metabolic dysfunction similar to AD that can increase susceptibility to viral infections, MS patients may experience higher rates of death during SARS-CoV-2 with COVID-19 (369). Treatment with metformin, commonly used during DM, can reduce the degree of functional impairment in obese individuals or those with DM during COVID-19 (52, 370). MS also may have common pathways with AD and Aβ (140, 252, 371). Tau seeding, also present in AD (7, 123, 311, 372–376), has been reported in the brains of MS patients (140) and this tau deposition may produce demyelination through injury to oligodendrocytes (128). Alterations in Aβ deposition similar to those observed in AD may also indicate early memory impairment in people with MS (371).

## 4 Addressing unmet clinical avenues for metabolic and neurodegenerative disorders

**Figure 2:**
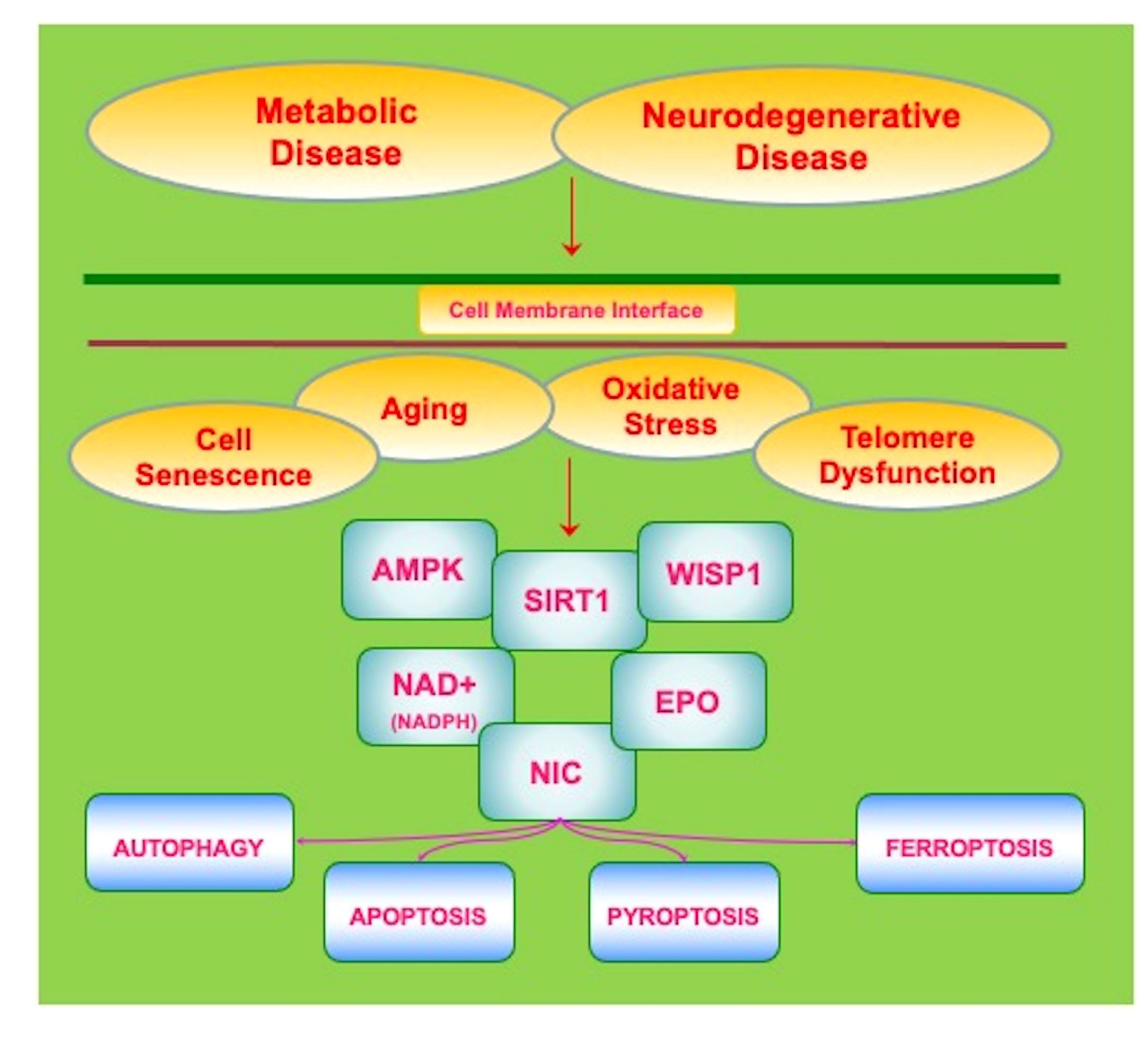
Innovative Avenues to Address Metabolic and Neurodegenerative Disease. Current therapies for metabolic and neurodegenerative disorders are unable to prevent the onset and progression of these disorders. The clinical course of these disorders is closely tied to intracellular process that are linked to aging-related disorders, telomere dysfunction, cellular senescence, and oxidative stress. Novel and innovative therapeutic strategies are needed to address metabolic and neurodegenerative diseases. Pathways that may offer new insights into these disorders involve the silent mating type information regulation 2 homolog 1 *(Saccharomyces cerevisiae*) (SIRT1), AMP activated protein kinase (AMPK), and Wnt1 inducible signaling pathway protein 1 (WISP1). These pathways are closely interconnected, can form complexes, and involve ß-nicotinamide adenine dinucleotide (NAD^+^), nicotinamide (NIC), and trophic factors such as erythropoietin (EPO). Ultimately these pathways serve to provide oversight of programmed cell death mechanisms that involve autophagy, apoptosis, pyroptosis, and ferroptosis as well as mechanisms that can lead to mitochondrial stress such as with nicotinamide adenine dinucleotide phosphate (NADPH) depletion.

Multiple factors can impact the role of metabolic disorders that can lead to the onset and progression of neurodegenerative disease. If one focuses upon metabolic disease and DM, therapies that improve nutritional intake that can be complemented by pharmaceutical agents to assist with serum glucose homeostasis and insulin resistance may limit periods of hyperglycemia and the complications of hypoglycemia (6, 8, 16, 19–21, 25, 27–29, 49, 167, 175, 176, 370, 377–379). Yet, progression of DM even at a less marked pace will ensue and can be affected by off-target treatment effects that result in cellular injury, neuronal and vascular cell loss, and the atrophy of organs (33, 87, 172, 380). In the nervous system, a number of diverse pathways that involve inflammation, infection, circadian rhythm, excitotoxicity, metabotropic receptors, tau, Aβ, mitochondrial injury, acetylcholine loss, heavy metal toxicity, and oxidative stress can lead to cognitive loss (24, 33, 34, 42, 50, 63, 65, 66, 96, 106, 119, 121, 131, 165, 168, 175, 205, 223, 226, 261, 282, 286, 303, 330, 338, 346, 381–398). Current therapies for AD that employ cholinesterase inhibitors may limit memory loss but these treatments do not stop the progression of disease (115, 164, 299, 399, 400). Recent developments for AD to use immunotherapy to decrease Aβ load in the brain also may reduce memory loss, but these therapies are currently limited to a small group of patients that are not at risk for cerebral microhemorrhages and also such treatments do not prevent overall disease progression (119, 126, 401). In regard to MS, disease modifying therapies (DMTs) can reduce the frequency of relapses in relapsing–remitting MS, but disease progression can continue (252, 362, 371). For example, despite the reduction in brain volume loss with DMTs, cognitive impairment can continue unabated (402). These treatment considerations that rest on the side of metabolic disorders as well as neurodegenerative disease warrant new avenues of inquiry for the development of innovative therapeutic strategies that may address the onset and progression of these disorders. Novel pathways that may offer new insights into these disorders involve programmed cell death regulation, the silent mating type information regulation 2 homolog 1 *(Saccharomyces cerevisiae*) (SIRT1), AMP activated protein kinase (AMPK), and Wnt1 inducible signaling pathway protein 1 (WISP1) (**Figure 2**).

## 5 Programmed cell death in metabolic and nervous system diseases

Programmed cell death that involves autophagy, apoptosis, ferroptosis, and pyroptosis has a vital role in the determination of both metabolic and neurodegenerative disorders (**Figure 2**). Recent studies on exome sequence analysis indicate that metabolic cellular dysfunction directly affects neuronal cell death through DNA and apoptosis (403). In metabolic disorders, programmed cell death can affect neuronal survival (24, 122, 310, 404–411), vascular integrity (8, 40, 61, 87, 182, 188, 412–414), mitochondrial function (42, 61, 172, 186), and inflammation (36, 50, 87, 186, 415–418). In a similar manner, programmed cell death in the nervous system can lead to neuronal and non-neuronal cell injury (65, 121, 132–134, 143, 148, 155, 224, 240, 262, 376, 405, 419–428), cerebral ischemia (91, 122, 182, 405, 423, 427, 429–433), microglial cell loss (120, 125, 133, 152, 356, 421, 430, 434, 435), and dysfunction of pathways for cognitive function (100, 120, 121, 141, 143, 148, 260–262, 275, 301, 421, 436–440).

During autophagy, organelles in the cytoplasm as well as other subunits in the cell are recycled for future remodeling of tissues (5, 121, 376, 408, 409, 428, 441, 442). Of the different forms of autophagy, macroautophagy is the prominent type of autophagy that is usually described and consists of sequestering proteins and organelles in the cytoplasm of cells into autophagosomes that will be merged into lysosomes that can be degraded and recycled (117, 136, 141, 275, 443). During microautophagy, components of the cytoplasm are sequestered for eventual digestion through invagination of lysosomal membranes (97). In chaperone-mediated autophagy, protein “chaperones” are created in the cytoplasm to carry components of the cytoplasm over the membranes of lysosomes (134, 136, 444, 445).

Autophagy can foster beneficial outcomes during metabolic and neurodegenerative disorders. Activation of autophagy can be necessary for fatty acid metabolism during obesity (62) and for the oversight of muscle tissue generation (441). Autophagy can oversee the proliferation and size of pancreatic β-cells (446), may limit insulin resistance during inflammation with high serum lipids in obesity models of autophagy *Atg7* gene deletion (447), may prevent diabetic nephropathy with maintenance of Atg7, Atg5, and LC3 autophagy proteins (448), and can prevent DM progression through promoting β-cell function and eliminating misfolded proteins and dysfunctional mitochondria (449). Increased physical activity in murine models helps control glucose serum regulation through autophagy pathways (450) that are tied to greater insulin efficacy (451) and improved function of microglial cells (416). In the brain, loss of autophagy activation can lead to memory impairment in AD with the progression of DM (44). The pathways that lead to the activation of autophagy may require inhibition of the mechanistic target of rapamycin (mTOR) with agents such as rapamycin or metformin (7, 49, 121, 284, 335, 359, 376, 393, 442, 452–457). In addition, activation of mTOR can inhibit autophagy induction through the phosphorylation of the autophagic related gene (*Atg*) protein Atg13 and UNC-51 like kinases (ULKs) such as UNC-51 like kinase 1 (ULK1) to prevent formation of the ULK-Atg13-FIP200 complex (97, 458). During mTOR inhibition and autophagy activation, reduction in ROS release occurs (459), dopamine cell survival is increased (460), neuronal demise is blocked through pathways of glutamine (461), and mitochondrial integrity is preserved (462). Autophagy activation can limit tau deposition (463) and reduce Aß accumulation with improved memory function and metabolic homeostasis (464). Control of blood mononuclear cells in MS during inflammation can be mediated by autophagy activation (465), induction of autophagy can improve the clinical outcome of relapsing-remitting and experimental autoimmune encephalomyelitis (466) and reduce retinal MS-induced degeneration (189), cytokine release and microglial activation is limited during autophagy activation in experimental autoimmune encephalomyelitis (358), and the risk for developing MS may be lessened during mTOR blockade and autophagy induction (359). Autophagy activity with metformin treatment also may lead to myelin repair with oligodendrocytes (356) and assist with the reduction of viral susceptibility during DM in over-weight individuals exposed to COVID-19 (52, 370).

However, there exists another side to autophagy that suggests careful modulation of activity is required for clinical disease. Cardiomyopathy (467), atherosclerosis (468), and endoplasmic reticulum stress (469) can ensue with advanced glycation end products (AGEs), elevated glucose exposure, and autophagy activation. Induction of autophagy can lead to the reduction in cardiac and liver tissue mass during treatments to improve glucose regulation (380), promote neuronal cell death under some conditions (470–472), prevent cerebral interneuron progenitor cell growth (473), result in mitochondrial injury (61, 145, 172, 216, 306, 379, 474–479), reduce numbers of progenitor endothelial cells (480), limit angiogenesis in the presence of elevated glucose (480), and lead to cognitive loss (141, 148, 275, 361, 393, 437). In addition, growth factor cell protection, such as with the trophic factor erythropoietin (EPO) (114, 304, 385, 481–484), requires decreases in autophagy activation in conjunction with modulation of mTOR, protein kinase B (Akt), the proline rich Akt substrate 40 kDa (PRAS40), and mammalian forkhead transcription factors to promote neuronal and vascular survival (145, 485–489).

Apoptotic cell injury can be initiated through pathways of oxidative stress (40, 68, 96, 120, 201, 216, 244, 245, 256, 260, 261, 303, 490–495) and inflammation (7, 36, 120, 143, 169, 212, 225, 256, 260, 275, 303, 310, 425, 430, 496–501) as part of metabolic and neurodegenerative disorders. Apoptotic cell death has early and late components that can occur in this process (97, 122, 408, 409, 426). The loss of PS membrane asymmetry is the early phase of apoptotic cell death (502–506). Once cells become injured, the PS residues become externalized on the cell membrane that attracts inflammatory microglia to recognize these injured cells and remove them from the central and peripheral nervous systems (502, 507–510). However, injured cells may recover if they are not engulfed by microglia. Treatments directed to restore PS membrane asymmetry for injured cells can then prevent microglial attraction and preserve the function of these necessary cells in the nervous system (66, 511–513). In contrast, the later phase of apoptotic cell death that consists of the destruction of nuclear deoxyribonucleic acid (DNA) (18, 96, 113, 229, 483, 514–518) and a cascade of caspase activation (65, 96, 130, 143, 347, 348, 405, 425, 483, 519) is not reversible.

Reductions in apoptosis activation can prevent cell injury during glial cell excessive activity and oxidative stress (120), limit dopaminergic cell demise during inflammatory cell activation (152), protect retinal cells during ischemia exposure (516), and increase neuronal cell survival during Aß toxicity (320, 520–522). Controlling apoptotic cell death also reduces inflammatory cell pathways (50, 186, 212, 225, 256, 260, 261, 418, 496, 497, 499, 500, 523–525) and can limit memory loss (7, 100, 143, 262, 421, 439). These pathways are significantly tied to microglial activity. Microglia account for about fifteen percent of the cells in the central nervous system and as noted can remove injured cells during apoptosis (97, 120, 133, 134, 152, 155, 425, 430, 502, 503). These inflammatory cells can release ROS to generate oxidative stress (7, 18, 62, 121, 282, 526–528) through pathways that involve Wnt signaling (2, 6, 97, 122, 181, 529–531), mammalian forkhead transcription factors (17, 67, 68, 117, 426, 519, 532), and growth factors with EPO (6, 145, 159, 482, 533–536). During cognitive dysfunction, microglial activity may lead to increased risk for the development of AD (141, 537) as well as endothelial dysfunction (119).

Ferroptosis is a process in the programmed cell death pathway that leads to the storage of iron in the cell that results in the inability to maintain glutathione homeostasis (225, 538, 539). Once oxidative defenses that require glutathione are lost, lipid peroxidation can ensue to result in the demise of cells (224, 229, 540). Ferroptosis can lead to cell death in multiple systems such as the musculoskeletal system (225), cardiovascular system (8, 540), and breast tissue (229). In the nervous system, ferroptosis may lead to cognitive impairment (7, 224, 274) and produce pathogenic T lymphocytes that lead to dysfunction in neuronal and glial cells (252, 538).

Given the associations of autophagy, apoptosis, and ferroptosis with inflammatory cell pathways, it is of interest to note that pyroptosis is part of the programmed cell death pathway that can specifically modulate inflammatory cell activity (5, 178, 410, 500, 541). The inflammasome, also known as the pyroptosome, is a supramolecular entity that initiates the pyroptotic cell death process. The inflammasome family of nucleotide-binding oligomerization domain and leucine-rich repeat-containing receptors (NLRs) has the members NLRP1, NLRP3, NLRP6, and NLRC4. Pattern recognition receptors responding to damage associated molecular pattern (DAMP) in host cells and pathogen-associated molecular pattern (PAMP) in families of microbes lead to the activation of inflammasomes and caspase 4, caspase 1, and caspase 5 (7, 303, 358, 515, 541–545). DAMP molecules with DNA and adenosine triphosphate (ATP) traverse through open cell membranes and lead to NLRP3 canonical inflammasome activation while caspase 5 and caspase 4 can result in noncanonical inflammasome activation with lipopolysaccharide proteins in infections with Gram-negative bacteria. For membranes to open, pores are formed through the degradation of N-terminal domain with the C-terminal domains as part of gasdermin proteins. Cytokines such as interleukin-1 family members are released to generate inflammatory reactions and require gasdermin since these cytokines cannot alone result in pore formation (2, 212, 515). Pyroptosis can result in cell injury as a result of cytokine release (410, 411) and lead to neuronal and vascular cell dysfunction that results in loss of memory and executive function (11, 60, 252, 263, 275, 298, 301, 344, 546). In addition, elevated cytokine release during pyroptosis can affect immune cell activity in the body (358) and lead to failed clinical outcomes, such as in MS patients, with elevated inflammasome levels (541).

## 6 SIRT1 regulation of cellular metabolism and neurodegeneration

The silent mating type information regulation 2 homolog 1 *(Saccharomyces cerevisiae*) (SIRT1) controls both cellular metabolism (11, 60, 111, 263, 275, 294, 298, 344, 400, 546–549) and neurodegeneration (98, 102, 117, 156, 210, 232, 240, 400, 438, 550–555). There exist mammalian homologues of Sir2 that are SIRT1, SIRT2, SIRT3, SIRT4, SIRT5, SIRT6, and SIRT7 (6, 12, 13, 97, 134, 255, 333, 556). SIRT1 exists in the brain, liver, heart, skeletal muscle, pancreas, adipose tissue, and spleen (16, 179, 210, 223, 440, 550, 557). SIRT1 is a histone deacetylase that controls transcription of DNA that involves acetyl group transfer from ϵ-N-acetyl lysine amino acids to DNA histones (6, 17, 70, 78, 106, 117, 223, 259, 309, 335, 558, 559) (**Figure 2**). Histone deacetylases oversee multiple cellular processes such as aging, wound healing, neuronal function, oxidative stress, transcription factor activity, cardiovascular function, and cancer (8, 78, 98, 175, 550, 560–563). One substrate for SIRT1 is the coenzyme ß-nicotinamide adenine dinucleotide (NAD^+^) (24, 42, 65, 70, 78, 96, 98, 111, 196, 330, 475, 550, 564).

SIRT1 regulation of cellular metabolic homeostasis can be critical to the onset and progression of disorders in the nervous system (13, 16, 17, 38, 98, 223, 259, 333, 544, 549, 565, 566). SIRT1 is dependent upon NAD^+^ and nicotinamide (24, 98, 210, 255, 417, 475, 514, 550). As a precursor for NAD^+^, nicotinamide is the amide form of vitamin B_3_ (niacin) (8, 34, 42, 330, 378, 567–570). Nicotinamide can be produced through SIRT1 transferring of the acetyl residue of the histone acetyllysine residue to the ADP-ribose moiety of NAD^+^. As part of a feedback pathway, nicotinamide can limit the activity of SIRT1 through the interception of an ADP-ribosyl-enzyme-acetyl peptide intermediate with the regeneration of NAD^+^ (571). As a result, nicotinamide can bind to sirtuins through NAD^+^ in the C pocket of sirtuins (572) and can noncompetitively inhibit SIRT1 (560) and prevent anti-inflammatory gene expression (573). In addition, SIRT1 activation through nicotinamide phosphoribosyltransferase (NAMPT) can occur with periods of glucose restriction. This leads to increases in NAD^+^ and reduction in nicotinamide levels that become ineffective to block SIRT1 (574). Replenishment of NAD^+^ can assist with cardiovascular health (53) with SIRT1 activation limiting inflammation, metabolic dysfunction, and cell injury (111, 113, 203). As an example during hyperglycemia, SIRT1 can increase vascular cell survival (575).

SIRT1 can oversee insulin sensitivity (8, 61, 78, 417, 576–578) and mitochondrial function (13, 34, 70, 240, 475, 558). SIRT1 expression is reduced in the liver and pancreas during high fat diets that can lead to insulin resistance (579). Elevated SIRT1 activity can modulate glucose and hepatic lipid processing to prevent metabolic syndrome dysfunction (580). SIRT1 controls insulin sensitivity via protein tyrosine phosphatase (PTP) (335, 581). SIRT1 also is a positive feedback system for insulin signaling through Akt and can lead to the activity of Akt through phosphotidylinositide 3-kinase (PI 3-K) (532, 581).

In the nervous system, SIRT1 activity can lead to neurite outgrowth and enhance neuronal survival in environments that limit nutrients (582). SIRT1 can foster survival for photoreceptor cells (583), prevent the senescence of endothelial cells (584), and enhance the function of mitochondria in embryonic stem cells during oxidative stress (585). The absence of SIRT1 activity may lead to dysregulation in the immune system such as during MS (406). SIRT1 activity may be required for limiting the toxicity of oxidative stress and preserving memory (262), fostering Aβ degradation (586), increase lifespan in higher level organisms (587), and protecting neuronal and vascular cells against oxidative stress (98, 134, 232, 240, 438, 502, 510, 550, 551, 554, 555, 588).

SIRT1 also functions through trophic factor regulation in metabolic and neurological disorders that is linked to NAD^+^ activity (34, 145, 203, 499, 533–535, 589). The trophic factor EPO employs SIRT1 to block depolarization of mitochondrial, release of cytochrome c, induction of BCL2 associated agonist of cell death (Bad) activation, and caspase cleavage (510). EPO through SIRT1 can protect neurons (590) that may be responsible for SIRT1 synaptic memory improvement (223). As a result of SIRT1 activity, EPO prevents mitochondrial injury (483, 521, 536, 591–593), increases microglial survival (594), blocks caspase activity (520), protects human cardiomyocytes (592), and oversees cellular metabolism (304, 482, 595, 596).

## 7 Oversight of metabolic and nervous system disorders through AMPK

AMP-activated protein kinase (AMPK) is an important component of the mTOR pathway, is intimately associated with SIRT1, and is a significant target for metabolic and neurodegenerative disorders (2, 30, 38, 112, 113, 118, 218, 229, 452, 558, 559, 597–600) (**Figure 2**). AMPK can lead to the generation of adenosine triphosphate (ATP), improve insulin sensitivity, oversee the oxidation of fatty acids, and reduce levels of oxidative stress (30, 34, 229, 230, 234, 288, 309). Increased AMPK activity is present in diets high in fish oil that can prevent endothelial cell injury (601) and improve insulin sensitivity (451). In the nervous system, AMPK can limit stroke damage in animal models of DM (602), reduce tau deposition (463), regulate neuroinflammation (134, 226, 603), reduce Aß brain accumulation (604), block Aß toxicity (605), oversee mitophagy with ULK1 (606, 607) and improve cognition in experimental models with DM and AD (608). Pain sensation that can become problematic with peripheral neuropathies in DM can be attenuated in experimental models with AMPK (292).

A number of agents that are involved in metabolic homeostasis also rely upon AMPK. Nicotinamide can protect mitochondria with the activation of AMPK (288). In addition, metformin and biguanides control autophagy through AMPK. DM cardiac cell injury through the activation of autophagy with AMPK is reduced during treatment with metformin (609). As previously noted, activation of autophagy under some circumstances can reduce toxicity from oxidative stress (33, 121, 145, 189, 216, 226, 306, 309, 540) and may shift to beneficial oxidative metabolism (610). In addition, other pathways such as Wnt family members may require AMPK activity to reduce neuronal brain injury (611). AMPK signaling is necessary with inhibition of mTOR pathways for the maintenance of electrical activity of the brain for control of behavior (597), for the acceleration of myelin brain recovery during treatment with metformin (356), and for mitochondrial preservation during ferroptotic cell death (229). Without AMPK activity, cell death, cell senescence, and mitochondrial loss can result (16, 113).

Bidirectional pathways of modulation of activity also exist for SIRT1 and AMPK. SIRT1 expression leads to deacetylation of serine-threonine liver kinase B1 (LKB1) that may be through indirect or direct means and can result in AMPK activation (612). Although AMPK does not directly activate SIRT1, SIRT1 activity can increase with AMPK either by elevating the cellular NAD^+^/NADH ratio that leads to deacetylation and downstream SIRT1 target activity changes to involve peroxisome proliferator-activated receptor-gamma coactivator-1α (PGC-1α) and forkhead transcription factors (613) or through increasing NAMPT to raise NAD^+^ and lower SIRT1 inhibitors such as nicotinamide (574). Resveratrol, an activator of SIRT1, can elevate AMPK activity through SIRT1 dependent or independent pathways (613, 614). Through combined pathways, AMPK and SIRT1 block mitochondrial loss (61) and limit endothelial cell death during elevated glucose exposure (575).

## 8 WISP1 in metabolic homeostasis and nervous system function

Closely coordinated with the pathways AMPK in metabolic and neurodegenerative disease is the Wnt1 inducible signaling pathway protein 1 (WISP1) (6, 75, 529, 615–618). WISP1 is a downstream target of the *wingless* pathway of Wnt proteins. Wnt proteins are cysteine-rich glycosylated proteins that can control cellular metabolism, stem cell proliferation, new vascular cell growth, musculoskeletal disease, nervous system sensation, and neuronal cell development (57, 83, 122, 181, 203, 346, 349, 350, 530, 531, 619, 620). Wnt signaling that includes Wnt1 can control autophagy (621–624), prevent endothelial cell death in experimental models of DM (412), limit dopaminergic neuron cell loss in PD (625), oversee repair of wounds during DM (626), assist with human β-cell proliferation (627), foster growth in the musculoskeletal system (57, 497, 628), and inhibit cognitive loss with DM and aging (629).

WISP1 is a CCN family member that consists of six secreted extracellular matrix associated proteins that are termed by the first three members of the family that include Cysteine-rich protein 61, Connective tissue growth factor, and Nephroblastoma over-expressed gene (130, 630) (**Figure 2**). WISP1 can be influenced by increased weight in humans, becomes elevated with insulin resistance in children and adolescents (616, 631), and is elevated during gestational DM (632). WISP1 may be vital for glucose homeostasis since it is over-expressed during regeneration of the pancreas (633), controls β-cell proliferation (75), and can control cellular senescence (634). WISP1 can stabilize atherosclerotic plaques (347) that can ultimately lead to cerebrovascular disease, can limit lipopolysaccharide-induced cell injury through pathways of Akt (348), can attenuate blood-brain barrier disruption (635), and decrease toxicity of oxidative stress and Aß exposure (97, 520, 522). However, it should be noted that WISP1, a trophic agent, also can promote tumorigenesis (281, 529, 636–642).

WISP1 is dependent upon the pathways of AMPK for glucose homeostasis and the ability to affect neuronal survival. WISP1 can oversee AMPK post-translational phosphorylation during cellular metabolism (6, 33, 455, 643–645). WISP1 controls the activation of AMPK activation by differentially decreasing phosphorylation of tuberous sclerosis 2 (TSC2) at serine^1387^, an AMPK target, and increasing phosphorylation of TSC2 at threonine^1462^, an Akt target (522). This process allows WISP1 to provide a minimal level of TSC2 and AMPK activity to offer a proper biological balance for optimum metabolic homeostasis and survival of cells. This balance in AMPK activation and levels is critical. Although AMPK activation can limit insulin resistance and oxidative stress (451) and assist with differentiation of adipocytes during lipid accumulation in obesity (646), under other conditions AMPK through autophagy may lead to cell injury. A fine balance of AMPK activity is necessary to increase basal autophagy activity and maintain neuronal and endothelial cell survival during periods of metabolic homeostasis loss (230, 234, 575, 584). AMPK can modulate apoptosis and autophagy during coronary artery disease (647) and ROS release (234, 648). This is also seen with the growth factor EPO. EPO controls AMPK during oxidative stress (522), inflammation (114, 385, 649), angiogenesis (650, 651), and modulation of endothelial nitric oxide synthase (652). The concentration of EPO and duration of treatment can influence a specific level of AMPK activity, as well as the activity of mTOR (114, 653–655). If this activity is not balanced, elevated EPO and AMK activity can lead to cell injury (656).

## 9 Discussion

With the increase in lifespan and NCDs, disorders of cellular metabolism that include DM and neurodegenerative disorders are increasing in prevalence throughout the world. These disorders may be significantly under diagnosed with estimates of at least thirty-five percent of individuals in developed countries not receiving appropriate care to slow the progression of metabolic and neurodegenerative disease. Metabolic and neurodegenerative disorders also impose a significant financial challenge for the treatment and care of individuals. It is expected that additional care costs for current unmet clinical and staffing needs will exceed over two trillion USD per year.

The clinical onset and progression of metabolic and neurodegenerative disorders is closely tied to aging, dysfunction in telomere processing, and the processes of cellular senescence and oxidative stress (**Table 1**). These underlying processes not only lead to neuronal and vascular death, mitochondrial loss, stem cell injury, and immune system dysfunction, but also result in significant co-morbidities in the nervous system leading autonomic dysfunction and peripheral neuropathies as well as cognitive loss. In fact, cognitive loss is now the seventh cause throughout the world for death and it is estimated that close to two hundred million individuals may have dementia by the year 2050. Although dementia may present in multiple neurological disorders, AD encompasses almost sixty percent of individuals with cognitive loss and it is estimated that more than sixty-five percent of people with MS have cognitive impairment. The cognitive loss in neurological disorders has an important metabolic basis and involves risk factors with APOE that can lead to the cognitive loss present in AD and MS as well as increase susceptibility to viral infections, such as during SARS-CoV-2 with COVID-19.

Metabolic disorders, such as DM, and neurologic disease remain a significant challenge for the treatment and care of individuals. Although strategies that address nutritional intake and the use of pharmaceutical agents to control glucose homeostasis can slow disease progression of DM, clinical off-target effects can lead to progressive neuronal and vascular cell loss and the atrophy of organs in the body. To a similar degree, present therapies for AD assist with symptoms of memory loss, but do not halt disease progression. In addition, recently approved therapies and DMTs for AD and MS that involve immunotherapies may be suited for a small subset of people, such as with AD, and can achieve some decrease in disease progression but the overall course of cognitive loss in these disorders will continue unabated. These clinical challenges to address the interplay between metabolic and neurodegenerative disorders require innovative strategies that can focus upon the underlying mechanisms of aging, oxidative stress, cell senescence, and cell death.

Programmed cell death pathways that involve autophagy, apoptosis, ferroptosis, and pyroptosis can play an important role in DM and neurodegenerative disorders. Autophagy can regulate the proliferation and size of pancreatic β-cells, reduce insulin resistance, prevent diabetic nephropathy, and limit progression of DM through promoting β-cell function and eliminating misfolded proteins and dysfunctional mitochondria. Autophagy activation in conjunction with mTOR inhibition can reduce ROS release, protect dopamine cells, preserve mitochondrial integrity, reduce inflammatory processes that may lead to clinical relapses, limit Aß and tau brain deposition, reduce cytokine release and microglial activity, and repair myelin in the nervous system. In regard to apoptotic cell death, strategies that can limit apoptosis activation can prevent cell injury during excessive glial activity during oxidative stress, limit ischemic toxicity to retinal cells, preserve dopaminergic cells during inflammation, and block cell demise during Aß exposure. Similar to apoptotic cell injury but involving iron accumulation in the cell with the loss of glutathione homeostasis, ferroptosis leads to cognitive impairment, immune dysfunction in T lymphocytes that can injure neuronal and glial cells, and cell death in the musculoskeletal system, cardiovascular system, and breast tissue. Pyroptosis cell death involves inflammasome activation that yields cytokine release, cell injury, inflammatory cell dysfunction, and neurovascular injury with cognitive loss.

Given that pathways of programmed cell death play a dual role in determining the fate of cellular survival and organ systems, it is important to recognize that a balance among these pathways is essential to optimize clinical outcome. For example, activation of autophagy can lead to cardiac disease, atherosclerosis, block interneuron progenitor cell growth, foster neuronal death, lead to memory impairment, and prevent neurovascular protection with growth factors. As a result, the basal activity of autophagy should be considered since changes in the autophagic flux have been shown to limit the induction of cell senescence. With apoptosis, it can be equally as crucial to control early apoptotic pathways that involve PS membrane asymmetry in an effort to block the later phase progression of the apoptotic cascade and prevent nuclear DNA degradation that leads to cell death. In addition, apoptotic pathways are tightly linked to inflammatory cell activity. Although increased glial cell activity may contribute to oxidative stress and apoptosis, microglial cells can be beneficial at times with autophagy induction to maintain cholesterol homeostasis (133), provide protection during amyotrophic lateral sclerosis (657), and for the clearance of amyloid in the brain (598). Triggering receptor expressed on myeloid cells 2 (TREM2) that can foster microglial survival can prevent inflammation during AD through forkhead transcription factors (FoxOs), Wnt signaling, and microglial activation (323, 658).

Pathways with SIRT1, AMPK, and WISP1 also provide us with clues for maintaining a proper environmental homeostasis for cells especially during aging processes that can lead to cognitive loss. SIRT1 is dependent upon NAD^+^ and nicotinamide and can control insulin sensitivity, prevent cell senescence, modulate immune system activity, promote Aβ degradation, and increase lifespan in higher organisms. In addition, mitochondrial oxidative stress can be dependent upon nicotinamide adenine dinucleotide phosphate (NADPH) depletion and increase of aldose reductase. These pathways are important in the pentose phosphate pathway that involves transaldolase, which is encoded by the *TALDO1* gene (659) and has been linked to Parkinson’s disease (660). Such mitochondrial dysfunction can be mediated through mTOR pathways that lead to antiphospholipid antibodies (661). Yet, feedback pathways are required at times through nicotinamide and Akt for replenishment of NAD^+^ and effective modulation of cellular pathways, such as those involving insulin signaling and inflammation. AMPK activation can improve insulin sensitivity, reduce oxidative stress toxicity, limit tau and Aß cell injury, and lead to cognitive improvement in conjunction with autophagy activation. Yet, during periods when autophagy activation can be detrimental, AMPK may require modulation to limit its activity. This loss of AMPK activity to alter autophagy pathways may negatively impact other pathways such as with SIRT1. There exist conditions when SIRT1 in conjunction with AMPK is required to prevent mitochondrial loss and vascular cell death. WISP1 also requires AMPK pathways to offer glucose homeostasis and protection of neuronal and vascular cells. The ability for WISP1 to effectively promote metabolic stability and cell survival is dependent upon WISP1 providing a proper biological balance of AMPK activity to foster cell survival, control inflammatory pathways, assist with growth factor protection, and prevent cell injury that can occur through autophagic and apoptotic mechanisms. The pathways of programmed cell death, SIRT1, AMPK, and WISP1 offer vital insights and are extremely attractive for identifying processes that can contribute to the onset and progression of metabolic and neurodegenerative diseases that can be linked to multiple entities such as APOE, SARS-CoV-2, NAD^+^, nicotinamide, and trophic factors, such as EPO, but will require further insight into the elaborate relationship of these pathways for effective clinical translation.

## Author contributions

KM: Conceptualization, Writing – original draft, Writing – review & editing.
